# The Interplay between Host Innate Immunity and Hepatitis E Virus

**DOI:** 10.3390/v11060541

**Published:** 2019-06-11

**Authors:** Yang Li, Changbo Qu, Peifa Yu, Xumin Ou, Qiuwei Pan, Wenshi Wang

**Affiliations:** 1 China-Malaysia National Joint Laboratory, Biomedical Research Center, Northwest Minzu University, Lanzhou 730030, China; y.li.2@erasmusmc.nl; 2 Postgraduate School Molecular Medicine, Erasmus MC-University Medical Center, 3015 CN Rotterdam, The Netherlands; c.qu@erasmusmc.nl (C.Q.); p.yu@erasmusmc.nl (P.Y.); x.ou@erasmusmc.nl (X.O.); 3 Department of Infectious Diseases, Molecular Virology, University Hospital Heidelberg, 69120 Heidelberg, Germany

**Keywords:** Innate immunity, Hepatitis E virus, Interferon, Inflammation

## Abstract

Hepatitis E virus (HEV) infection represents an emerging global health issue, whereas the clinical outcomes vary dramatically among different populations. The host innate immune system provides a first-line defense against the infection, but dysregulation may partially contribute to severe pathogenesis. A growing body of evidence has indicated the active response of the host innate immunity to HEV infection both in experimental models and in patients. In turn, HEV has developed sophisticated strategies to counteract the host immune system. In this review, we aim to comprehensively decipher the processes of pathogen recognition, interferon, and inflammatory responses, and the involvement of innate immune cells in HEV infection. We further discuss their implications in understanding the pathogenic mechanisms and developing antiviral therapies.

## 1. Introduction

Hepatitis E virus (HEV) represents an emerging zoonotic pathogen imposing a growing health burden [1]. Globally, it has been estimated that 2.3 billion people have possibly encountered this virus, whereas the clinical outcomes vary dramatically among different populations [2]. In general, HEV infection is self-limiting, but can cause severe diseases in special patients. Acute infection in pregnant women bears high risk of triggering acute liver failure (ALF), leading to a high mortality rate, and most of these patients are from resource-limited regions infected with the non-zoonotic genotype 1 HEV [3,4]. In European countries, chronic infection has been widely described in immunocompromised patients, in particular, organ transplantation patients [5,6]. Chronic infection is mainly caused by the zoonotic genotypes 3 [5] and 4 [7]. Evidently, the host immune responses are critical in determining the distinct outcomes of HEV infection among the different individuals [8].

The host immune system, including innate and adaptive immunity, essentially protects against pathogen invasions, but over-activation or dysregulation may cause pathogenesis [9]. As the first line of host defense, the innate immune system rapidly, but non-specifically, responds to viral infections. Upon sensing the pathogen, the host produces a large spectrum of cytokines and chemokines, in particular, the antiviral interferons (IFNs). IFNs trigger the transcription of hundreds of interferon-stimulated genes (ISGs), as the ultimate antiviral factors to combat the infection or further regulate immune response. The pro-inflammatory cytokines and chemokines induce an inflammatory response and recruit other immune cells to coordinately orchestrate the antiviral status [9,10].

The quantity and quality of host immune responses essentially determine the infection course of HEV and the clinical outcome in patients. The response of host innate immunity to HEV infection has been widely observed in experimental models and in patients. In turn, HEV has been reported to evade host immunity through multiple sophisticated strategies. Here, we aim to in depth decipher the dynamic interplay between HEV and the host innate immunity.

## 2. Innate Immune Response to HEV Infection

### 2.1. Recognition by Pathogen-Recognition Receptors in HEV Host Cells

The innate immunity controls and restricts the spread of infection immediately after contacting with the pathogen. The innate immune system detects viral infection through the recognition of pathogen-associated molecular patterns (PAMPs) by pathogen-recognition receptors (PRRs). These PAMPs are specifically present in the pathogen or generated during infection [11,12]. There are three distinct classes of PRRs in recognizing viruses, including toll like receptors (TLRs), retinoic-acid-inducible gene I (RIG-I)-like receptors (RLRs), and NOD-like receptors (NLRs). TLRs sense virus on cell membrane or in endosomes, whereas RLRs and NLRs detect virus in cytoplasm.

**Figure 1:**
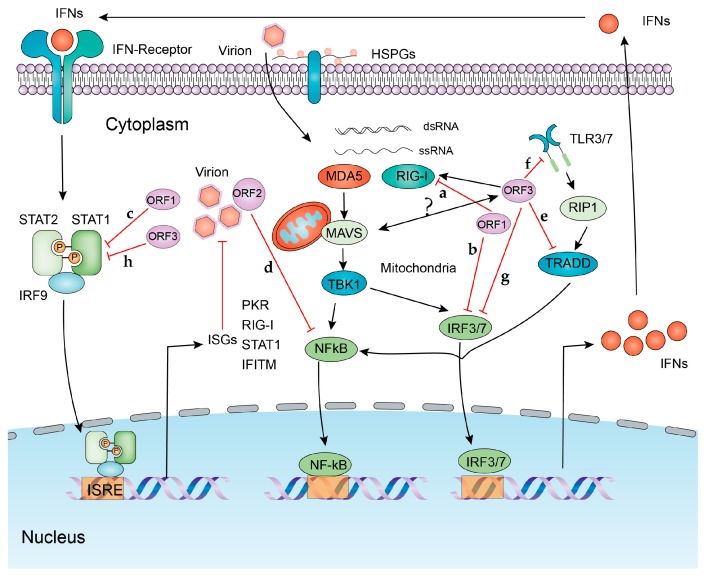
The interplay of the interferon (IFN) antiviral signaling and HEV. Upon infection, HEV is recognized by the host pattern-recognition receptors, including MDA5, RIG-I, TLR3, and TLR7. This triggers their downstream cascades, including IRF3 and NF-κB, leading to the production of IFNs. Subsequently, IFN binds to the receptors and results in phosphorylation of STAT1 and STAT2. Phosphorylated STAT1 and STAT2 form a complex with IRF9 (ISGF3). This complex translocates to nucleus and binds to IFN-stimulated response element (ISRE) to induce the transcription of antiviral IFN-stimulated genes (ISGs). RIG-I and MDA5 have been identified to inhibit HEV replication. On the other hand, HEV has evolved strategies to evade host IFN response. ORF1 inhibits phosphorylation of STAT1 and IRF3 as well as deubiquitination of RIG-I. ORF2 inhibits activation of NF-κB. ORF3 blocks phosphorylation of STAT1 and IRF3, but also has been reported to induce IFN production through increased activation of RIG-I. a, b, c, d, e, f, g, and h indicate the important interactions between viral proteins and host factors.

TLRs are cell membrane-associated PRRs that are present on the cell surface or in the endosomes. They can recognize viral nucleic acids or other components to trigger the production of IFNs and other cytokines through different cascades, such as TLR3 recognizing dsRNA, whereas TLR7/8 recognizes ssRNA [13,14,15]. TLRs are abundantly expressed in hepatocytes, the primary target cells of HEV. TLRs serve as principal elements of the hepatic immune system that defend infections in general. Both TLR3 and TLR7 signaling have been found to recognize hepatitis B (HBV) and C (HCV) virus to trigger an innate antiviral response [16,17,18,19]. In cell culture models, overexpression of TLR3 inhibits HEV replication. Consistently, blocking the TBK1/IKKε complex, the downstream molecules of TLR3 enhances HEV replication [20] (Figure 1). TLR signaling is roughly divided into two distinct pathways based on the usage of either MyD88 or TRIF adaptor molecules. Suppression of both MyD88 and TRIF inhibits TLR3-mediated IL-8 induction by HEV [21]. MyD88 deficiency attenuates the induction of IL-6 and RANTES by HEV. However, there is no effect of TRIF knockdown on IL-6 levels, suggesting that TLR2 and TLR4 are also involved in sensing HEV [21]. Interestingly, ORF3 of HEV augments the poly (I:C)-induced TLR3 pathway and enhances TBK1-mediated IFN-β expression [22]. In pregnant women infected with HEV, the expression levels of TLR2, TLR3, TLR4, and IFN-γ are significantly higher in those who recovered from acute infection compared to the ones that developed ALF [23]. Consistently, down-regulation of TLR3 and TLR7, as well as TLR downstream molecules, has been observed in another cohort of HEV infected pregnant women with ALF [3]. Thus, both in vitro and in vivo data support the prominent role of TLRs in recognizing HEV and triggering antiviral and inflammatory responses. 

RLRs include melanoma differentiation associated gene 5 (MDA5), retinoic acid-inducible gene (RIG-I), and laboratory of genetics and physiology gene 2 (LGP2), and all are located in cytoplasm [24,25]. Short dsRNA and 5′-triphosphate RNA are ligands for RIG-I, while MDA5 mainly recognizes long dsRNA [24,26]. Both MDA5 and RIG-I consist of two caspase activation recruitment domains (CARDs) at their N-terminal domains, a helicase domain, and a C-terminal domain. Upon binding to foreign RNA, the CARD domain of MDA5 and RIG-I recruits mitochondrial antiviral signaling (MAVS, also known as IPS-1, VISA, or Cardif) [27,28,29,30] to activate IRF3/7 and NF-κB, leading to the production of IFNs and other cytokines. In hepatocytes, RIG-I recognizes the 5′-ε region of HBV pgRNA and the polyuridine motif of HCV genome 3′ non-translated region, whereas HCV replicative dsRNA is recognized by MDA5 to initiate IFN-related antiviral responses [31,32,33,34,35]. However, whether MDA5 and RIG-I represent bone fide sensors for HEV remains to be clarified, although infection of HEV has been found to upregulate the expression of both genes. Earlier studies have suggested that MDA5 and RIG-I may recognize HEV RNA in human hepatic cells (Figure 1) [20,36]. However, a recent study has demonstrated that loss of MDA5 or/and RIG-I in mouse embryonic fibroblasts does not affect HEV RNA-induced IFN response [37]. Whether this discrepancy is attributed to the different cell types remains to be further investigated.

Besides hepatitis, HEV infection is also associated with a broad spectrum of extra-hepatic manifestations in patients including neurological diseases [38,39,40], renal injuries [41], and acute pancreatitis [42,43]. In experimental models, the direct infection of non-hepatic cell types, including neurons, brain microvascular cells, and kidney cells, has been demonstrated [44,45,46]. In pregnant women, HEV can also result in severe placental diseases, and the direct infection has been recently demonstrated at the human maternal–fetal interface. Genotype 1 HEV efficiently replicates in the tissue explants of decidua basalis and fetal placenta and stromal cells, and importantly triggers the production of a panel of cytokines and chemokines [47]. Thus, the innate sensors in these non-hepatic cells are also likely able to recognize HEV but require future research to further clarify.

### 2.2. Innate Immune Cells on HEV Infection

A viral infection rapidly recruits innate immune cells such as natural killer (NK) and natural killer T (NKT) cells to the site of infection [48]. Upon recognition, NK cells secrete cytokines including IFN-γ and tumor necrosis factor-α (TNF-α) to modulate antiviral immune responses [49]. NK and NKT cells constitute a major fraction of lymphocytes in the liver, and they are particularly important in the pathogenesis of viral hepatitis [50]. Both cell types play a critical role in the induction of liver cell injury during acute hepatitis B and C [51,52]. It has been suggested that NK cells may also mediate immune response to HEV infection [53]. In patients with acute hepatitis E, the percentages of NK and NKT cells among peripheral blood mononuclear cells (PBMCs) are significantly lower, whereas the proportions of activated NK and NKT cells are higher compared with those in uninfected controls [54]. This is consistent with another report that HEV infection is particularly severe in pregnant women with a decrease in NK cell activity [55]. The decreased percentages of NK and NKT cells in circulation may be explained by increased migration of these activated cells to the liver or apoptosis induction because of activation. However, CD56 (natural killer cells) and Granzyme B (activated NK cell marker) are significantly increased in biopsies from ALF patients caused by hepatitis E, compared with normal livers [56], and CD69 (activated NK cell marker) expression is increased in immunocompromised organ transplant patients acutely infected with HEV [57]. This might be associated with enhanced cytotoxic activity [58]. Mast cells constitute another major sensory arm of the innate immune system. In Mongolian gerbils, HEV infection increases the number, but also the activation, of mast cells in the liver and small intestine [59].

### 2.3. IFN and Inflammatory Responses to HEV Infection

Upon recognition of PAMP, PRRs activate intracellular signaling cascades, ultimately triggering IFN and inflammatory responses. Among the three types of IFNs, type I and III IFNs activate the same pathway involving the signal transducer and activator of transcription 1 (STAT1) and signal transducer and activator of transcription 2 (STAT2) [60]. Phosphorylated STAT1 and STAT2 form a complex with IRF9, also known as IFN-stimulated gene factor 3 (ISGF3). ISGF3 translocates into the nucleus and binds to IFN-stimulated response element (ISRE) to activate the transcription of hundreds of ISGs [61,62]. ISGs are the genetic effectors coordinating antiviral and immunomodulatory activities [9,10,63].

In cell culture, inoculation with infectious HEV particles activates type III, but not type I IFN response, with transcription of multiple ISGs. This requires MAVS or MDA5, and to a lesser extent RIG-I [64]. The low infectivity of HEV in cell culture models often fails to adequately mimic the robust viral replication in patients, because the viral load in patient blood can reach up to 10^7^ copies/mL [65]. A subsequent study delivering in vitro generated HEV genomic RNA into cells has demonstrated a robust induction of type IFN I response. This is independent of MDA5, RIG-I, and MAVS, but requires the downstream transcription factors IRF3/7 [37]. As discussed, the role of MDA5 and RIG-I in sensing HEV remains not fully clear [20,36,37], but their anti-HEV activities as ISGs have been convincingly demonstrated. By profiling the known antiviral ISGs, MDA5, RIG-I, and IRF1 have been identified as the most potent anti-HEV effectors [66]. RIG-I inhibits HEV replication by further induction of antiviral ISGs, partially through the JAK-STAT cascade, but without requirement of IFN production [66]. IRF1 inhibits HEV replication through induction of the expression and phosphorylation of STAT1. This triggers the transcription of many antiviral ISGs, but without the production of IFNs [67]. ISG15 is an IFN-induced ubiquitin-like protein with anti- or proviral functions depending on the context [68,69]. HEV infection has been demonstrated to induce ISG15 expression both in cell culture and pig models [70]. Interestingly, loss of ISG15 has no major effect on HEV replication but sensitized the response to IFN treatment. This is probably related to the enhanced expression of other general antiviral factors, including PKR, MX1, and OAS1 [70].

Inflammatory responses coordinated by the secretion of various cytokines and chemokines protects against infection, but dysregulation may cause serious pathogenesis. The production of inflammatory cytokines upon HEV infection has been widely observed in cell culture [21], animal models [71,72], and patients [23,73]. In HEV-infected pregnant women compared with non-pregnant women, the levels of TNF-α, IL-6, and IFN-γ are significantly higher [73]. This is in line with the observation that ex vivo culturing of PBMCs from acute hepatitis E patients without ALF produces more IFN-γ and TNF-α, compared to that from patients who developed ALF [23]. Collective evidence has indicated a central role of TNF-α and the NF-κB pathway in HEV triggered inflammatory response. In cell culture, TNF-α has been shown to moderately inhibit HEV replication. Interestingly, it can induce a subset of ISGs via the NF-κB cascade to synergize the anti-HEV effect of IFN-α [74].

### 2.4. The Implications of Innate Immune Response in Patient Outcome and Therapeutic Development

Exposure to HEV in healthy individuals usually results in asymptomatic infection. This likely reflects a strong host immune response against the infection, but such mechanisms are difficult to be captured and studied in detail. In asymptomatic blood donors, the role of anti-HEV antibody development and clearance has been shown [75]. In an Egyptian cohort, cell-mediated immune response has been indicated in asymptomatic individuals [76]. In symptomatic acutely infected patients, it is apparent that the innate immunity essentially contributes to the self-limiting process.

During pregnancy, the maternal immune system is dramatically altered to protect the fetus, such as the expression level of cytokines are changed to mediate the immunological tolerance [77]. This has been proposed as a key mechanism causing severe outcomes upon HEV infection, while many details remain unknown [78]. Differences in the expression of TLRs, production of inflammatory cytokines, and the immune cell compartment have been observed comparing HEV infected pregnant women that developed ALF compared to those without ALF or the non-pregnant population. Speculatively, this dysregulation may favor the infection and concurrently cause more severe liver damage, resulting in ALF and even death. In organ transplant patients, the use of immunosuppressive medication has been widely recognized as the key mechanism and cause of chronic hepatitis E [79,80]. These drugs primarily target the adaptive immune cells, in particular T cells, to prevent organ rejection. However, they are also able to affect innate antiviral effectors, and this at least, in part, contributes to the development of chronic infection [81].

As a potent antiviral cytokine, IFN-α has been approved for treating chronic hepatitis B and C for decades. In cell culture [82] and animal models [83], IFN-α exerts substantial anti-HEV activities. In chronic hepatitis E patients, several retrospective case series and case reports have documented an efficacy, but this is often associated with severe adverse events, including graft rejection and thrombocytopenia [82,84,85,86,87]. Nevertheless, understanding the anti-HEV mechanisms of IFN response will open new scenarios for developing more specific antiviral strategies. For instance, a number of MDA5 or RIG-I agonists are at various phases of preclinical or clinical development for treating viral infections [88]. These agents may exert more potent but specific antiviral efficacy alleviating the concerns of side effects.

## 3. Viral Strategies to Counteract Innate Immune Response

Despite the robust host response to HEV infection, the virus has developed many strategies to counteract and evade the defense mechanisms. Current knowledge mainly deciphers the interactions between HEV viral proteins with the host innate immunity.

ORF1 encodes a non-structural polyprotein, including several functional regions (Met, Y, PCP, HVR, X, Hel, and RdRp), which are essential for HEV replication. ORF1 has been shown to inhibit IFN-β expression induced by poly (I:C) transfection in cell culture [89]. This is mainly attributed to the Papain-like cysteine protease domain (PCP) and macro domain (X) that are responsible for inhibiting the activation of RIG-I and TBK-1 as well as the phosphorylation of IRF3 (Figure 1) [89]. The cooperation of methyltransferase (Met) and PCP has been shown to inhibit the ISRE promoter activity and the expression of ISGs through inhibiting nuclear translocation and phosphorylation of STAT1 [90]. Interestingly, the X domain is able to inhibit the secretion of ferritin in cell culture [91]. As an acute-phase protein, ferritin is abundantly secreted in HEV infected patients associated with inflammatory response. Thus, the X domain has been proposed to attenuate host immune response by preventing ferritin secretion [91]. Finally, the RNA-dependent RNA polymerase (RdRp) domain has been reported to interact with miRNA to facilitate HEV replication [92].

ORF2 encodes the capsid protein, which binds 5′ end of the genome, and is involved in viral encapsidation [93]. Three forms have been identified, including the infectious, glycosylated, and cleaved ORF2 [94]. However, only the infectious ORF2 is assembled into viral particles [94] and mediates entry [95]. ORF2 has been shown to inhibit NF-κB activity by inhibiting IκBα ubiquitination (Figure 1) [96]. The host transmembrane protein 134 (TMEM134) interacts with ORF2 to attenuate its inhibitory effect on NF-κB by ORF2 [97]. ORF2 can also impair the host’s apoptotic response to favor HEV infection [98].

ORF3 as a multifunctional protein has been proposed to create a favorable environment for HEV infection and pathogenesis [99,100,101]. In respect to the regulatory effects on innate immunity, it has been shown to inhibit STAT1 phosphorylation and ISG expression upon HEV infection (Figure 1) [102]. ORF3 of genotype 1 has been reported to downregulate the expression of tumor necrosis factor receptor 1-associated death domain protein (TRADD) and receptor-interacting protein kinase 1 (RIP1), thus inhibiting TLR3 mediated activation of NF-κB upon poly (I:C) treatment [103]. In line with this, ORF3 inhibits the expression of endogenous IFNα/β through inhibiting the expression of TLR3 and TLR7 [99]. ORF3 also inhibits LPS-induced cytokines and chemotactic factors [56]. It has been further demonstrated that ORF3 inhibits the activation of NF-κB JAK-STAT and JNK-MAPK pathways induced by TNFα, IFN-γ, and Anisomycin, respectively [99]. Counterintuitively, another study has demonstrated that ORF3 enhances IFN production upon poly (I:C) treatment through increased activation of RIG-I in a genotype-dependent manner [22]. Overall, ORF3 has been widely demonstrated to inhibit IFN and inflammatory responses to shape a favorable cellular environment for HEV infection.

Recently, ORF4 has been found in genotype 1 HEV. It has been reported to interact with RdRp to facilitate HEV replication [104], but whether and how ORF4 regulates host immune response remains unknown and deserves to be explored.

## 4. Conclusion and Perspectives

A growing body of evidence has revealed the active interactions between the host innate immunity and HEV infection. Upon infection, a spectrum of receptors and/or sensors are able to sense HEV to induce IFN and inflammatory responses. This essentially establishes the antiviral states, but dysregulation may cause serious pathogenesis, such as in pregnant women and organ transplant patients. In turn, HEV has developed sophisticated strategies to counteract the host innate immune response, mainly through the interactions with the viral proteins. Thus, the interplay between host immunity and the virus eventually deters the infection course and decides the clinical outcome.

There remain several knowledge gaps to be further investigated. Firstly, the exact receptors/sensors that recognize HEV and the subsequent cascades in initiating responses are not fully clear yet. Secondly, the mechanisms of how the dysregulation of immune and inflammatory responses causes pathogenesis remains largely elusive. Moreover, the clinical relevance of many observations coming from the overexpression of viral proteins in different cell systems remains to be established. Further understanding these insights shall help to reveal the detailed mechanisms of causing hepatic and extra-hepatic diseases, and to device therapeutic and interventional strategies to combat HEV infection.
